# Supplementary figures and images for: Isotopic signatures induced by upwelling reveal regional fish stocks in Lake Tanganyika
Source: PLoS One. 2023 Nov 8;18(11):e0281828. doi: 10.1371/journal.pone.0281828 (PMC10631627; doi:10.1371/journal.pone.0281828)

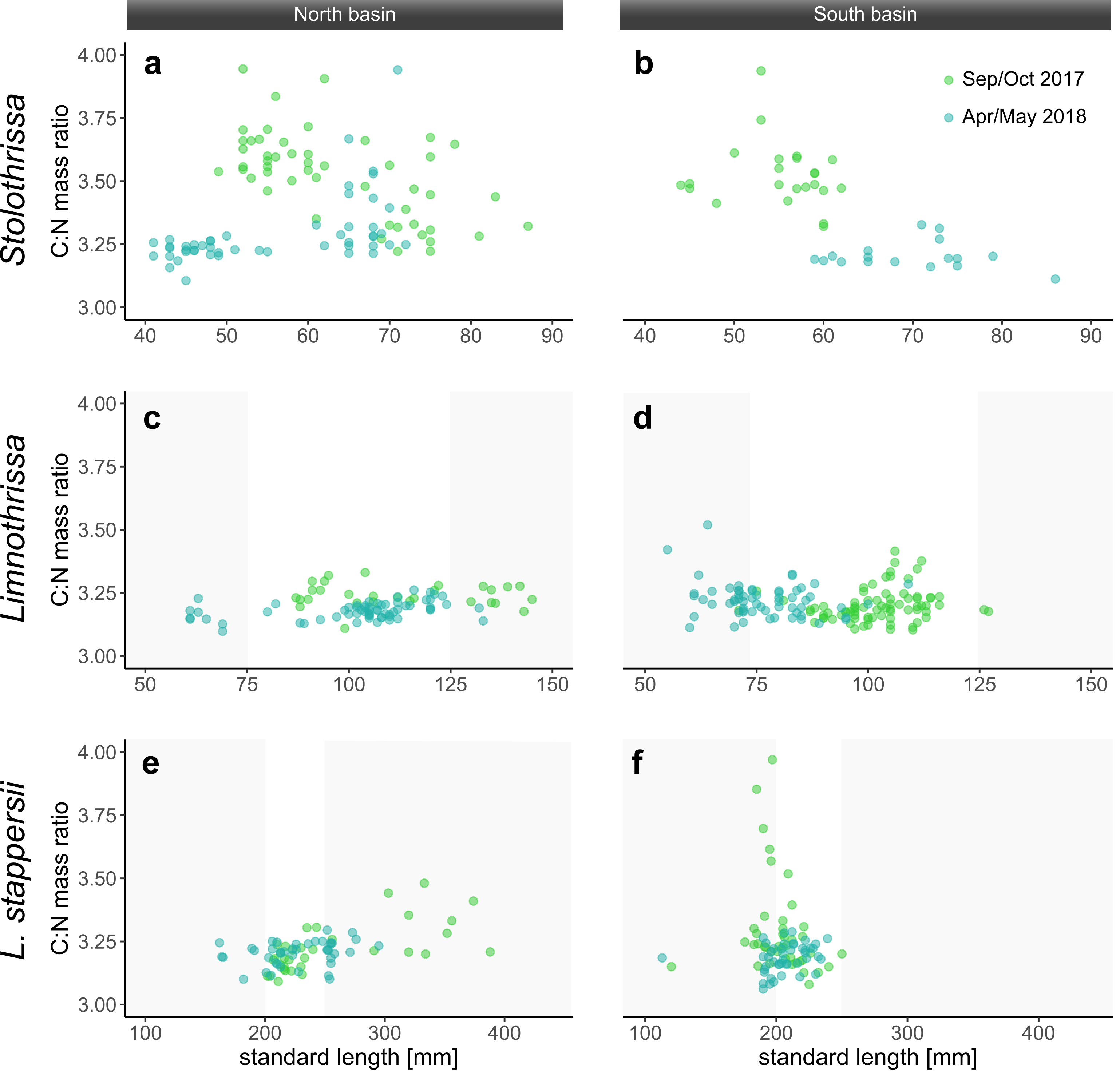

Supplement: S1 Fig — C:N mass ratio of (a,b) Stolothrissa tanganicae, (c,d) Limnothrissa miodon, and (e,f) Lates stappersii versus standard length in the northern and southern basins during the end of the dry season and the end of the rainy season. Only stations 1, 2 (north) and 7, 9 (south) are depicted. The shaded areas mark the 50 mm cut-off range for the population comparisons used in Fig 6. (TIF) [file pone.0281828.s001.tif]

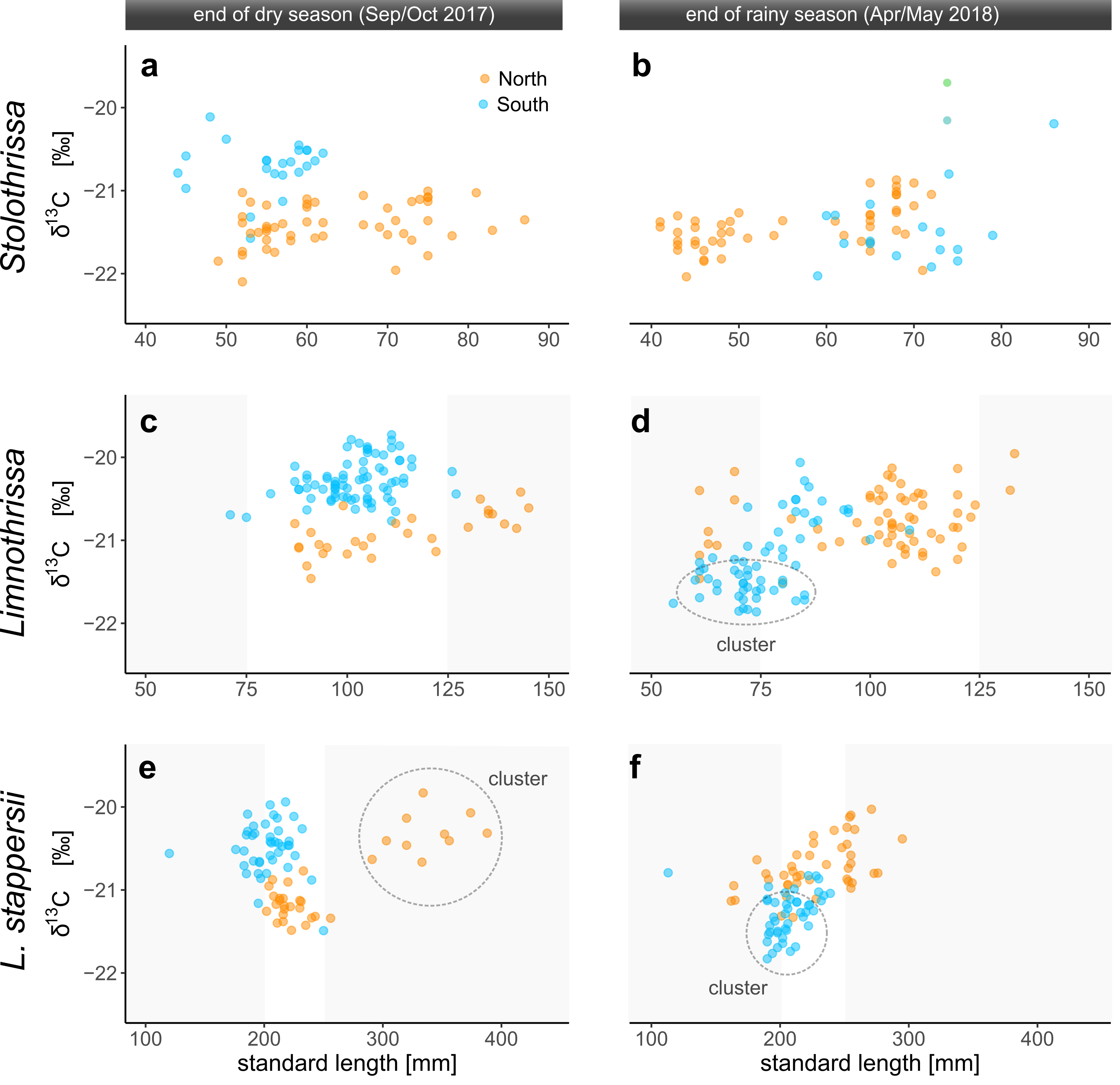

Supplement: S2 Fig — C:N corrected [56] δ13C of (a,b) Stolothrissa tanganicae, (c,d) Limnothrissa miodon, and (e,f) Lates stappersii versus standard length for the end of the dry season and the end of the rainy season. Only stations 1, 2 (north) and 7, 9 (south) are depicted. The shaded areas mark the 50 mm cut-off range for the population comparisons used in Fig 5. The sampled populations of L. miodon and L. stappersii from the end of the rainy season (d,f) were characterized by dense clusters of observations within a narrow size and δ13C range which may have skewed the basin-scale comparisons. (TIF) [file pone.0281828.s002.tif]

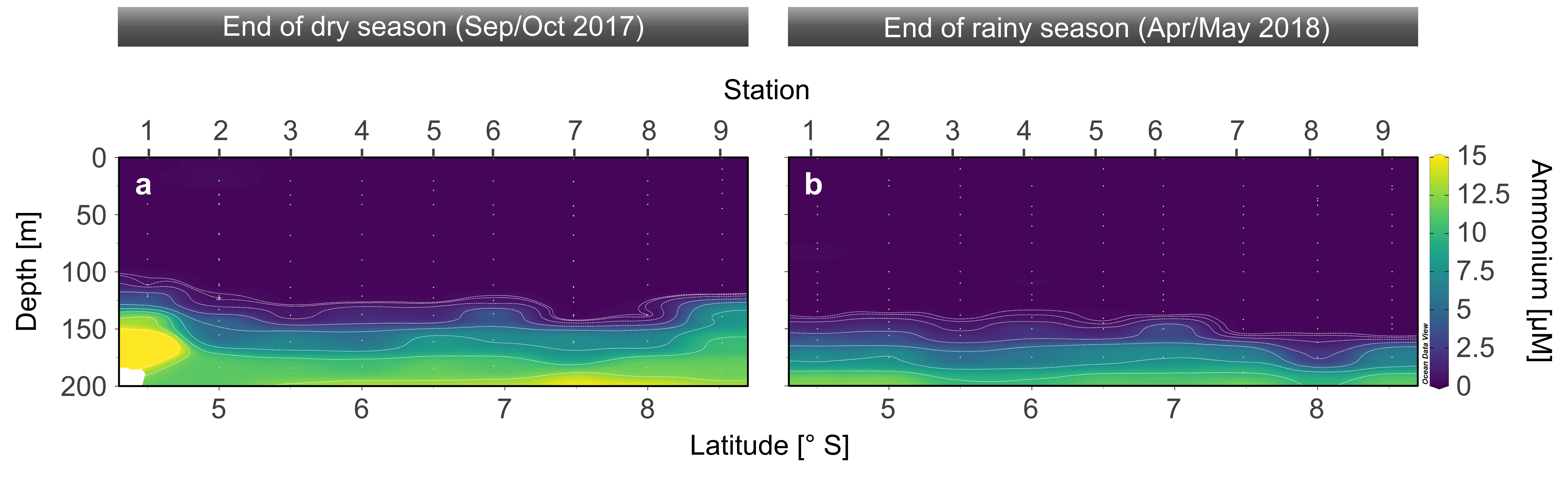

Supplement: S3 Fig — (TIF) [file pone.0281828.s003.tif]

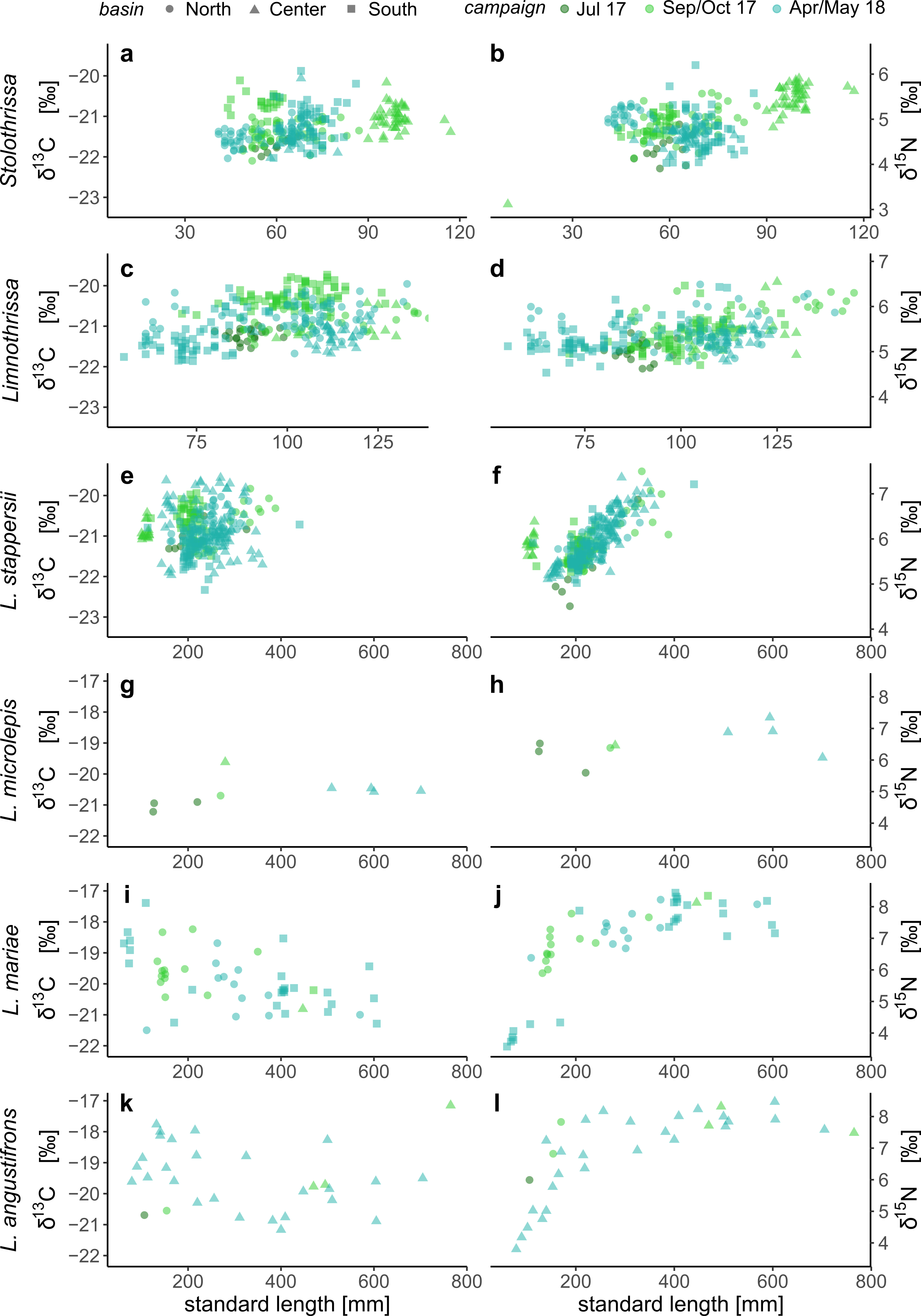

Supplement: S4 Fig — C:N corrected according to Post et al. [56] δ13C (left) and δ15N (right) of (a,b) Stolothrissa tanganicae, (c,d) Limnothrissa miodon, (e,f) Lates stappersii, (g,h) Lates microlepis, (i,j) Lates mariae and (k,l) Lates angustifrons versus standard length including all sampling locations and campaigns. Samples from the central basin and July 2017 were included for completeness, but were not included in the north-south and seasonal analysis presented in Fig 5. Note the different y-axis scaling. (TIF) [file pone.0281828.s004.tif]

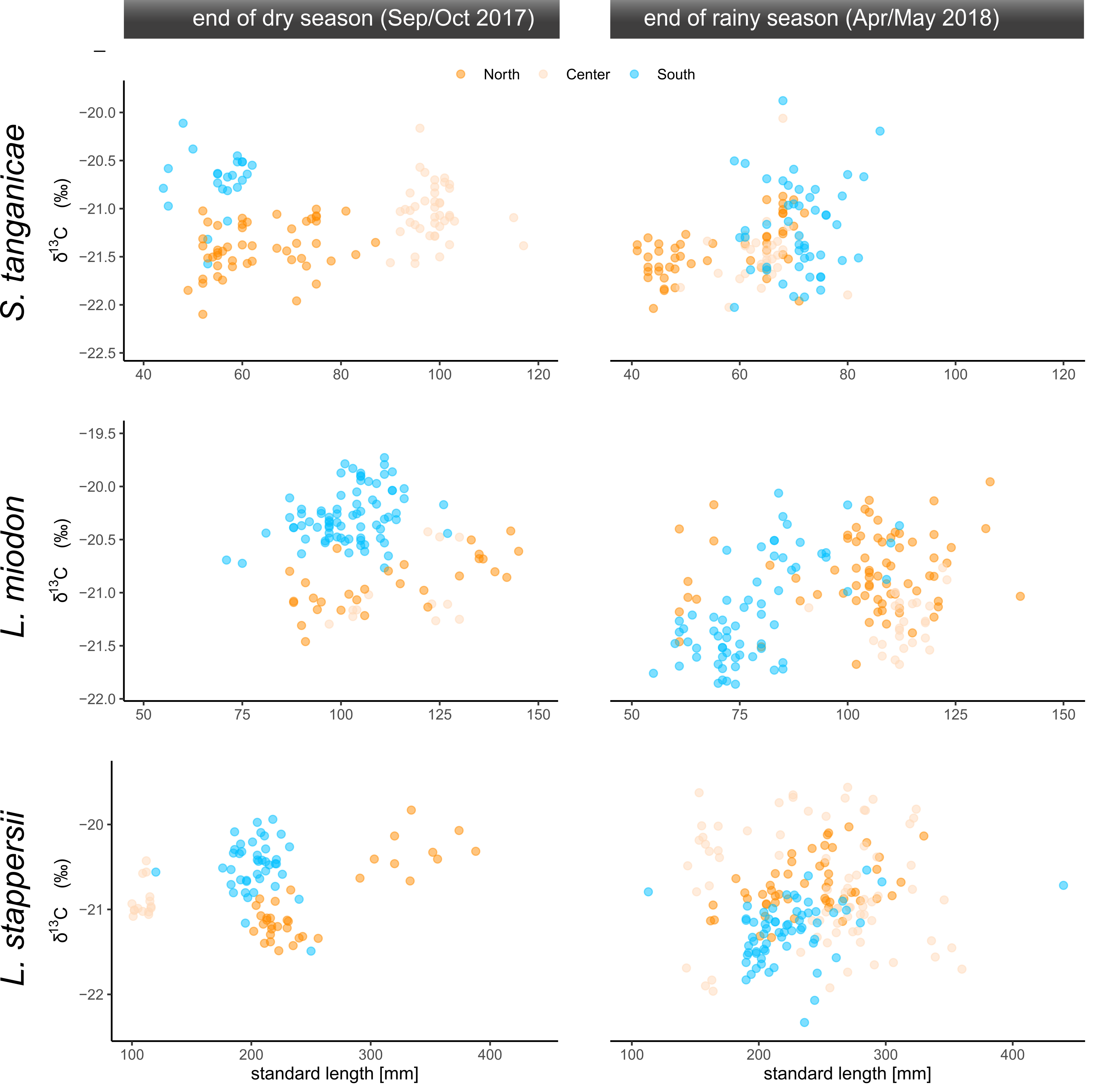

Supplement: S5 Fig — Carbon (normalized for C:N mass ratio according to Post et al. [56]) stable isotope signatures of Stolothrissa tanganicae, (c,d) Limnothrissa miodon, (e,f) Lates stappersii, including samples from the central basin, at the end of the dry season (left) and the end of the rainy season (right). (TIF) [file pone.0281828.s005.tif]

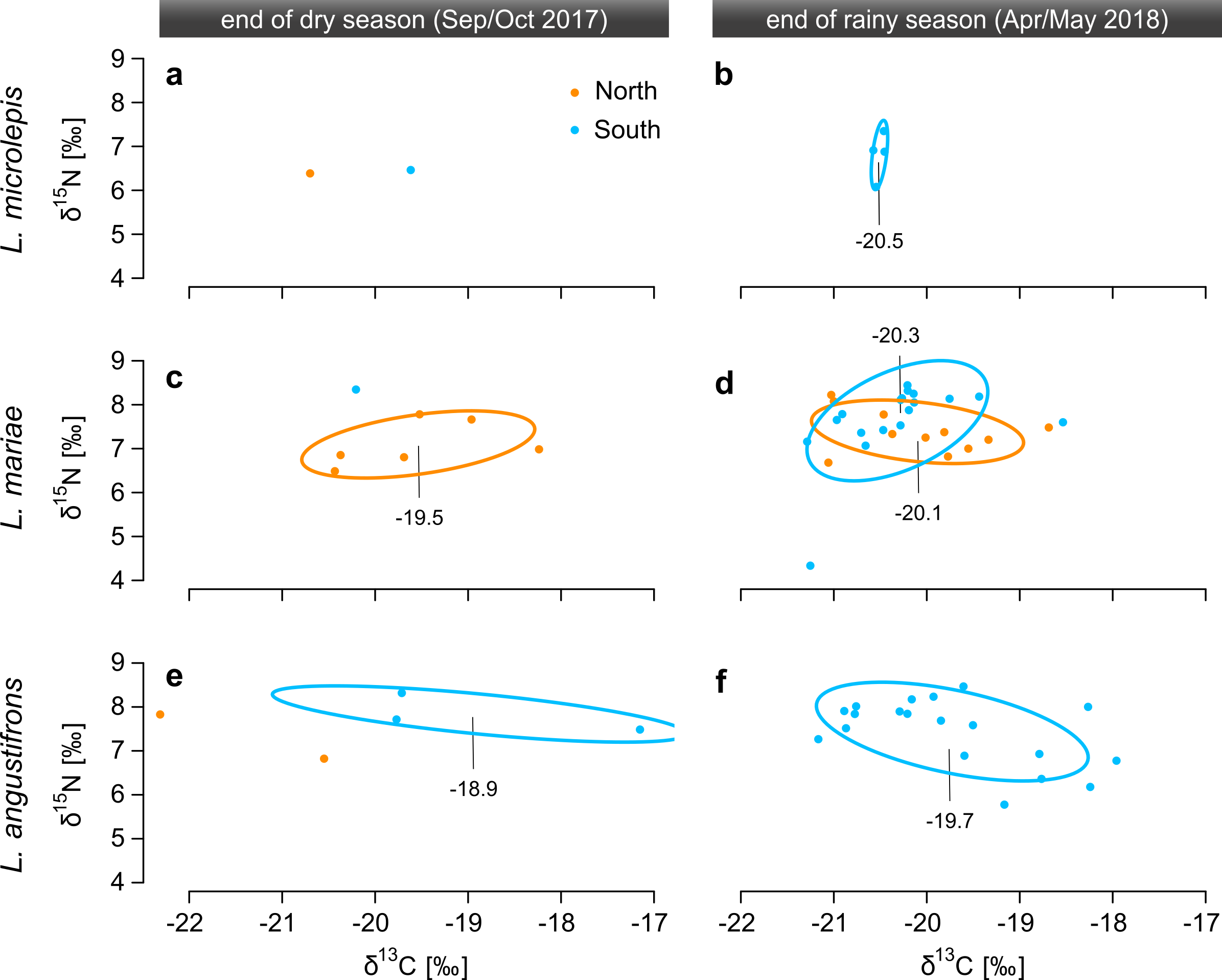

Supplement: S6 Fig — Carbon (normalized for C:N mass ratio according to Post et al. [56]) and nitrogen stable isotope signatures of the large Lates species, namely (a,b) Lates microlepis (c,d) Lates mariae, and (e,f) Lates angustifrons at the end of the dry season (left) and the end of the rainy season (right). Orange dots represent the northern basin (stations 1–3) and blue dots represent the southern basin (stations 7–9). Numbers indicate the mean δ13C of a population. Only individuals >150 mm were included in this analysis to reduce ontogenetic effects on the isotope signatures. (TIF) [file pone.0281828.s006.tif]

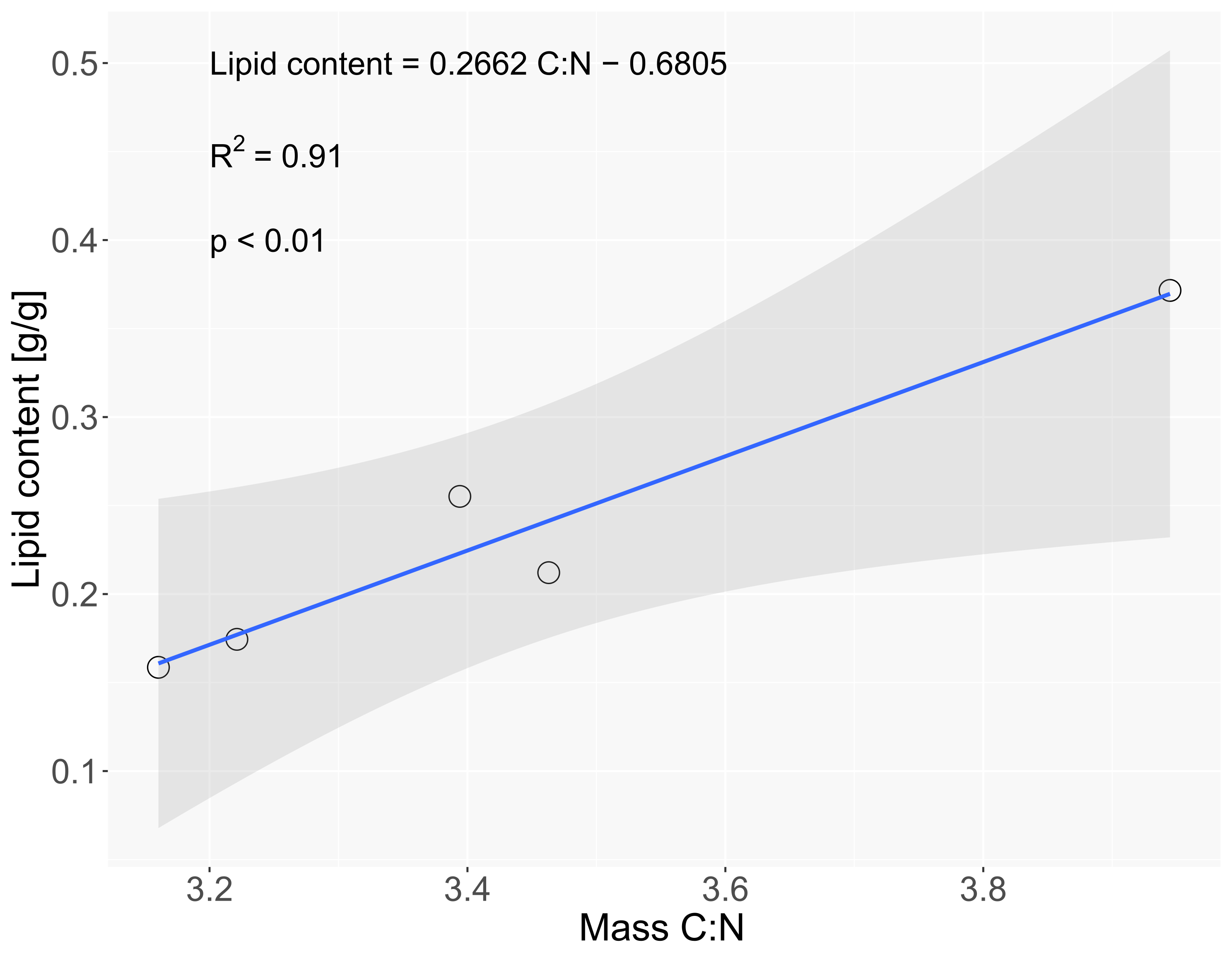

Supplement: S7 Fig — Each dot represents a tissue sample from one specimen. (TIF) [file pone.0281828.s007.tif]

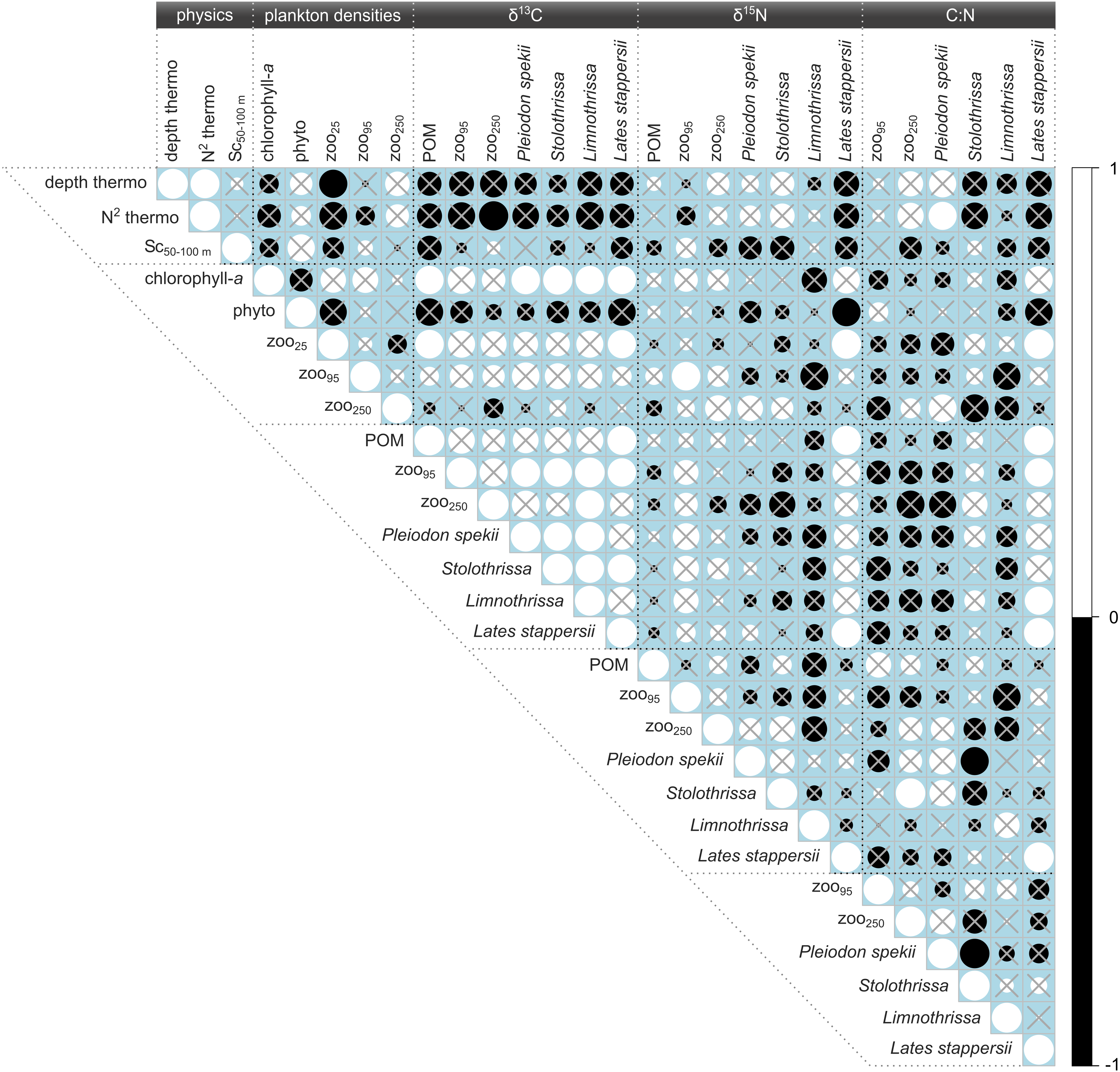

Supplement: S8 Fig — We selected the five stations across the north-south transect with the highest overlap among all variables (stations 1, 2, 6, 7, 9; S2 Table). Insignificant correlations (p > 0.05) are marked by grey crosses. Depth thermo: depth of the primary thermocline; N2 thermo: buoyancy frequency of the primary thermocline; Sc50-100 m: Schmidt stability of the 50–100 m depth interval; Phyto10 μm: phytoplankton abundance of the >10 μm size fraction; Zoo25/95/250 μm: zooplankton parameters of the >25, >95, or >250 μm size fractions. (TIF) [file pone.0281828.s008.tif]

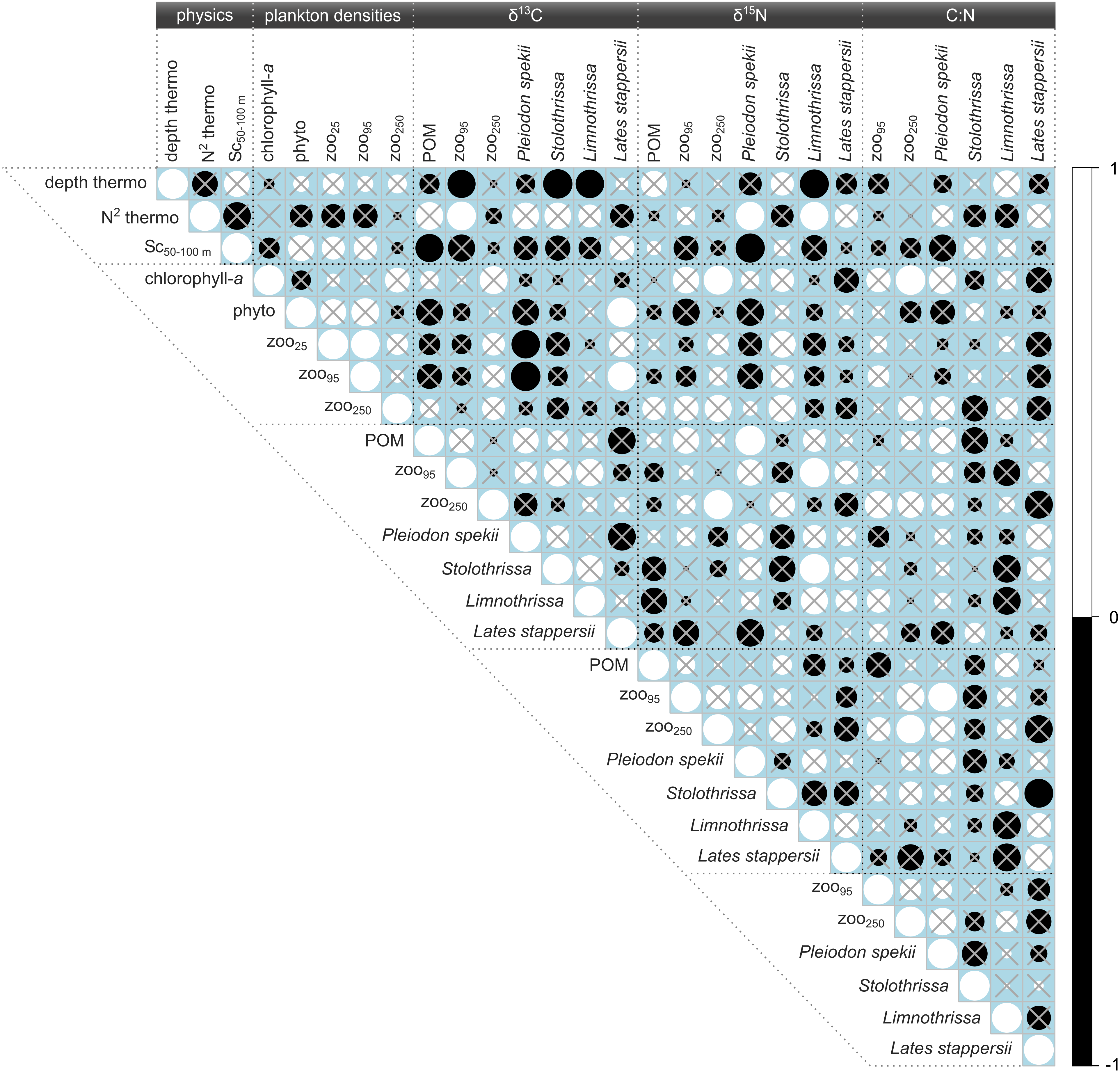

Supplement: S9 Fig — We selected the five stations across the north-south transect with the highest overlap among all variables (stations 1, 2, 4, 7, 8; S2 Table). Insignificant correlations (p > 0.05) are marked by grey crosses. Depth thermo: depth of the primary thermocline; N2 thermo: buoyancy frequency of the primary thermocline; Sc50-100 m: Schmidt stability of the 50–100 m depth interval; Phyto10 μm: phytoplankton abundance of the >10 μm size fraction; Zoo25/95/250 μm: zooplankton parameters of the >25, >95, or >250 μm size fractions. (TIF) [file pone.0281828.s009.tif]

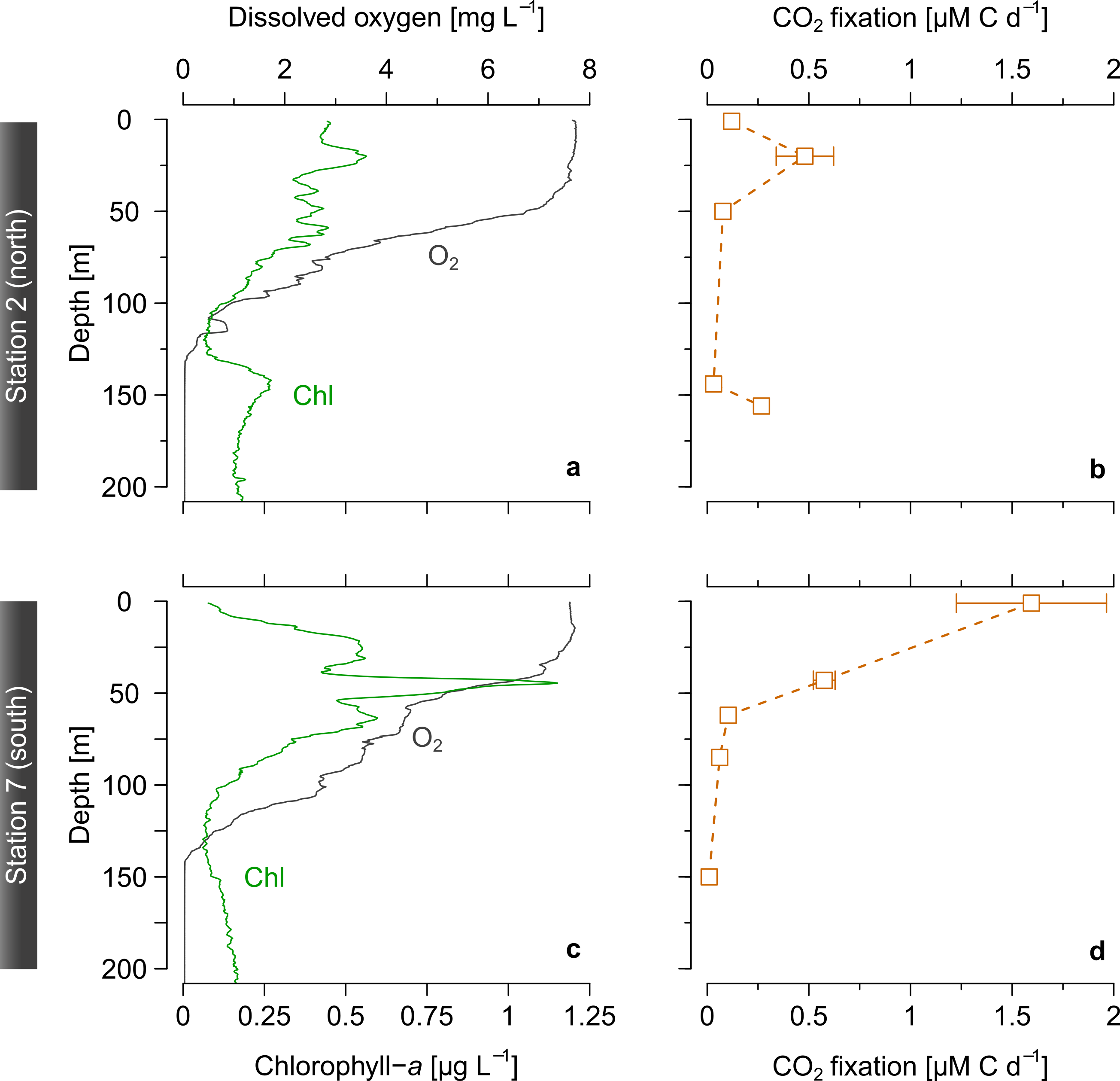

Supplement: S10 Fig — (a,c) Oxygen and in-situ chlorophyll-a as well as (b,d) CO2 fixation rates from stations 2 in the north (a,b) and 7 in the south (c,d). (TIF) [file pone.0281828.s010.tif]

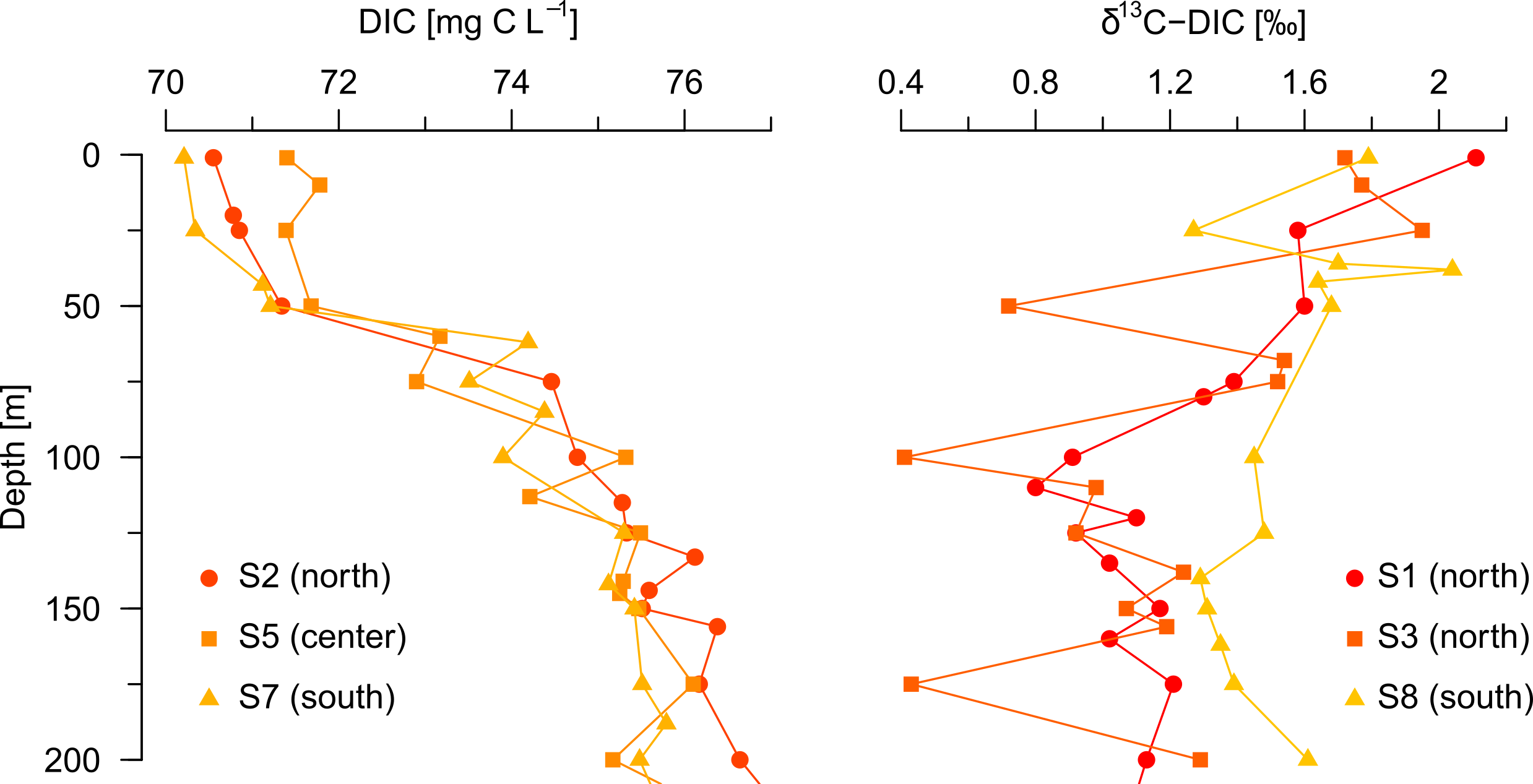

Supplement: S11 Fig — (a) DIC concentration profiles from stations 2, 5, and 7. (b) δ13C-DIC profiles from stations 1, 3, and 8. (TIF) [file pone.0281828.s011.tif]

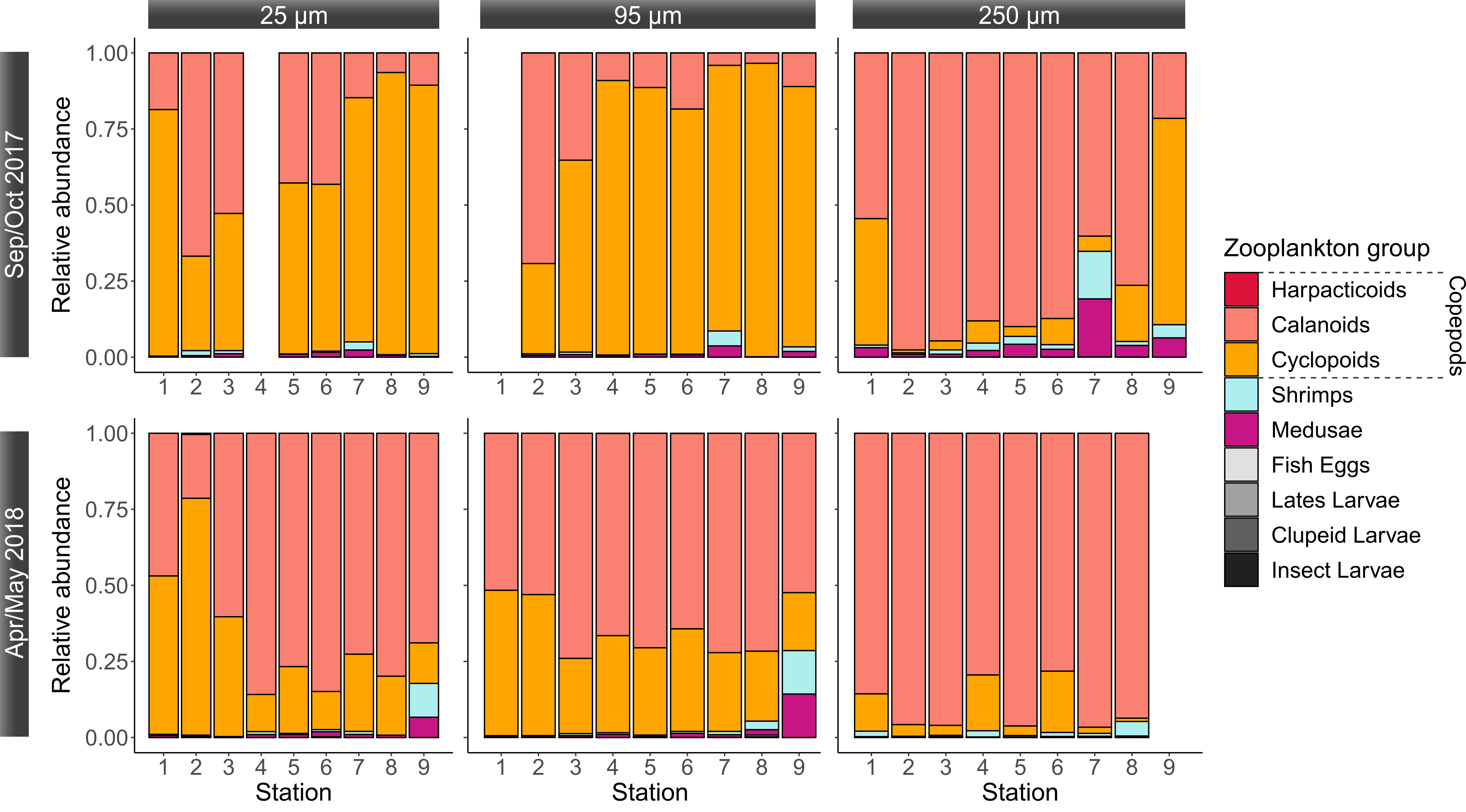

Supplement: S12 Fig — (TIF) [file pone.0281828.s012.tif]

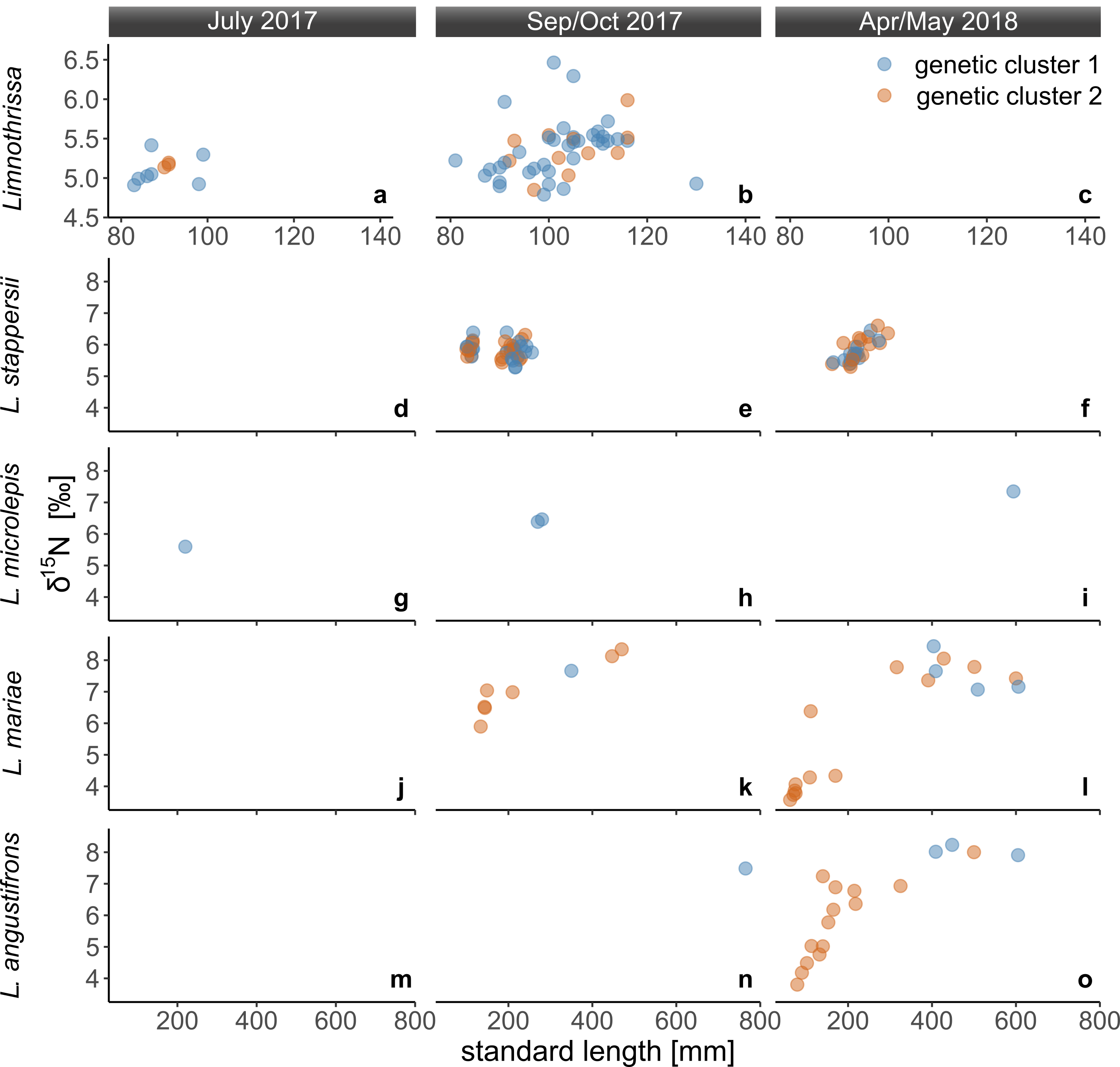

Supplement: S13 Fig — (a-c) Limnothrissa miodon, (d-f) Lates stappersii, (g-i) Lates microlepis (j-l), Lates mariae, and (m-o) Lates angustifrons. Note the different axis scaling between Limnothrissa and the Lates species. (TIF) [file pone.0281828.s013.tif]
