# Supplementary material for: Isotopic signatures induced by upwelling reveal regional fish stocks in Lake Tanganyika
Source: PLoS One. 2023 Nov 8;18(11):e0281828. doi: 10.1371/journal.pone.0281828 (PMC10631627; doi:10.1371/journal.pone.0281828)
Supplement: Attachment — Submitted filename: Response to Reviewers.docx [file pone.0281828.s016.docx]

Below is a point-by-point response to all reviewer comments. Changes in the manuscript are documented in the mark-up mode version of the revised manuscript.

**Reviewer #1:** This manuscript concerns measurements of stable carbon and nitrogen isotopes in fish tissues from Lake Tanganyika. Seasonally varying hydrology, with respect to patterns of upwelling and nutrient availability are hypothesized to affect changes to fish trophic structure – and thus community ecological function. A suite of limnological and biogeochemical parameters were measured to contextualize isotope measurements. The statistical analysis was sound, however sample sizes for some ellipses were quite small. This value wasn’t interpreted heavily though. Overall, interpretation is sound.

*REPLY: Agreed. Also considering the suggestions from the other reviewer and the editor, we analyzed our data set in a more comprehensive fashion. The key pieces are the two correlation matrixes for both seasons (see figures 7, S7, and S8). These correlations yielded consistent results that we think are useful in interpreting the key patterns emerging from the dataset. Even though not all relationships that we refer to were statistically significant, they were consistent, systematic, and unsurprising from a causal point of view. The test parameters are discussed in L.695-700:*

*“Despite the congruent patterns across the food web, it is not surprising that most correlations were statistically insignificant, given our sample size of five and the chosen Spearman's rank method. Rank-based tests sacrifice explanatory power in favor of not assuming normal distribution.”*

*More information underlying all analyses, including the mean values, standard deviations, and sample sizes, are now compiled in supplementary tables (see S1 and S2 tables). Even though we agree that discussing some statistical parameters, such as sample size, in more detail would be worthwhile, we did not discuss those deeper in the manuscript. This decision grounds on two reasons:*

1) *Our analyses revealed significant north-south differences in δ^13^C for all organisms with large sample sizes (3 ≤ n ≤ 74), i.e.* P. spekii *and the three major fish species (see figure 5) and clear, but not significant north-south differences for phyto- and zooplankton with lower sample sizes (n ≤ 3). We think that these systematic and coherent differences are sufficiently convincing for the qualitative conclusion that we draw: there is a significant north-south deviation in δ^13^C across all trophic levels linked to the dry season upwelling/mixing. We do not draw quantitative conclusions about more extent of particular factors on the absolute isotope values, i.e. trying to find the true mean of a distribution, which would require a more in-depth statistical analysis.*
2) *In connection to the qualitative nature of our argumentation – and given the length and complexity of the manuscript – we prioritized discussing context that is important for understanding the causal links on which we found our argumentation (e.g. DIC dynamics, primary productivity rates, tissue turnover times).*

**Reviewer #2:** This paper study patterns of stable isotopes across the food web in Lake Tanganyika when the whole lake is stratified and when water is circulating resulting in differences in stratification and upwelling between the north and south parts of the Lake. The results suggest that differences in isotopic signal traverse the food web when there is water circulation but not when the lake is stratified. One conclusion of this pattern is that pelagic fish use “regional grounds” for foraging that should be accounted for in management.

Although there are no direct flaws in the paper it is very hard to read. Mainly the result section is very long and difficult to grasp the important information and I suggest to put detailed numbers and differences into a Table or Supplement. The result section could almost be summerised in: at the end of the rainy season no differences and at the end of dry season there was differences in the isotoic signal that was tracked through the food web.

*REPLY: Done. We streamlined the entire manuscript according to this suggestion, with most revisions focusing on the results chapter. Most prominently, we rewrote and shortened the hydrodynamics and biogeochemistry sections (3.1) and moved auxiliary results including the respective figures (formerly figures 3 and 4) to the supplements, i.e. DIC concentration and isotopic composition, CO_2_ fixation rates, and the zooplankton community composition.*

*In addition, we compiled the data in a summary table and additional calculated a statistical test across the data set (see figures 7, S7, and S8 as well as tables S1 and S2). We believe that this analysis not only improves readability, but also provides stronger support for the conclusions we drew.*

I also have some concern regarding the title and the conclusion about “regional fish population”. The fish populations pick up the dC signal of their food but that does not mean they are “regional” (this is a bit problematic in itself as it is not defined what is meant, maybe causing a confusion). As far as I understand the isotopic signal does not reveal anything about where they spawn or how they move in the lake. Of course they don’t swim back and forth on a weekly basis but I don’t see any evidence against that a fish is in one part of the lake in one period and 7 month later in the other part, it will just pick up the current regional isotope signal. It would be informative to know how, when and where these fish species spawn, pelagic, demersal, littoral spwners, buoyant eggs? Although I can find it likely with some kind of regional population structure of these fish species I cannot see the evidence against it.

*REPLY: Correct. Our genetic results do provide evidence that there is lake-wide gene flow at sufficiently long time scales (i.e. there is no notable genetic structure at the lake basin scale). Thus, by “regional populations” we mean regional on a basin-wide and seasonal scale (see abstract and the conclusion from section 4.2 in the discussion: L.701-716). There are several life history mechanisms which could reconcile these findings. For example, there could be substantial movement during early life stages that would act to genetically homogenize populations, followed by relatively little movement at later life stages.*

*We agree that information on spawning locations would provide relevant context. Although knowing spawning ground locations would be interesting, we do not think that the spawning grounds will affect our results due to the chosen size ranges, which exclude juvenile fish from our analysis (see section 2.9). In support of this argument, north-south differences in δ^13^C during the dry season were consistent across all trophic level, whereas differences with respect to spawning ground preferences exist among the studied fish species. In brief,* Stolothrissa *and* Lates stapppersii *spawn offshore, whereas* Limnothrissa *and the three larger* Lates *species,* L. microlepis, L. mariae, *and* L. angustifrons *nearshore (see e.g. Coulter, 1970, 1991; Matthes, 1967).*

*Given the length and complexity of the manuscript, we therefore chose not to include this information in the manuscript.*

Specific comments
Abstract
Poorly written with many odd sentences, e.g. l. 26 (global warmin related), l. 31 (differences in habitat), l. 36-38 (regional forage grounds and record…gradients), l. 39-40 (regional population on a seasonal…), l 41 those?,

*REPLY: We improved the phrasing throughout the abstract to make links clearer and achieve more coherence.*

l. 42-43 Not part of this study at all,

*REPLY: We integrated the genetic results from the previous publications within this project here. Drawing conclusions about the time span in which the fish populations can be regarded as “regional”, was only possible by combining the genetic results with the additional findings from the isotope data. Those two sets of data provide information on the distribution of the fish over long (genetics) or relatively short (isotopes) timescales. Additionally, we incorporated the existing genetic data into new ways here, i.e. we analyzed whether they correlate with the isotope data (see Supplementary Figure S11; formerly Fig. S7).*

l. 45-46 you don’t show these at all,

*REPLY: See Supplementary Figure S11 (formerly Fig. S7). Nonetheless, we have removed the respective sentence from the abstract, because it was not a main result.*

l. 48 how does basin relate to regional?

*REPLY: Those are the same. We have replaced “basin-wide” or “basin-scale” with “regional” throughout the abstract to establish more coherence and avoid confusion.*

Methods:
Reference or motivation to statement on l. 258.

*REPLY: Collecting fresh fish samples via net hauls and doing all the biogeochemical measurements and experiments at the same time was logistically not feasible. Thus, the “fish team” collected fish samples from local fishermen and processed the samples on-board, while the water column samples were retrieved and processed.*

*The information that local fishermen usually fish within a 20 km radius from their landing sites is expert knowledge gained from long-term interviews and monitoring efforts by local TAFIRI researchers. There is no published reference which we could cite in this context.*

l. 295. Fish was sampled from landing sites, not the sampling stations.

*REPLY: Correct. We changed “stations” to “sites” or “landing sites adjacent to station …”. For simplicity and coherence, we refer to all sampling sites as “station” thereafter.*

l. 303. In general are mixing models used to infer the diet of consumers from stable isotopes, but that has not been applied here, or? If not, why? Wouldn’t this show if they differ in diet between northern and southern area just not isotope signal?

*REPLY: We chose not to apply isotopic mixing models for three reasons. First, the δ^15^N of potential prey organisms – the bulk zooplankton community – varied by up to 6.7 ‰ within the basins and maximum values were similar to the highest fish δ^15^N values. Second, the δ^15^N values of individual zooplankton taxa can vary greatly, with reported values ranging from 0.1 to 5.9 ‰ in Lake Tanganyika. Third, exact trophic discrimination factors are not known for Lake Tanganyika, but appear to largely deviate from the norm. Together these factors impede setting up a mixing model to quantify the trophic position of the fish.*

*We rephrased L.725-738 accordingly to better convey these arguments in the manuscript. Thereafter, we chose to interpret only the absolute δ^15^N values to a level, that the context of our data allows (L.739-749).*

Results
Far too long and detailed, see above.
l. 435 Compare with l. 415-418. Don’t seem to match to me.

*REPLY: According to the new analyses and figures (see figures 4, 7, S7, and S11, as well as tables S1 and S2), we rewrote the entire section, which we believe is more consistent now (see section 3.2).*

Discussion:
l. 569-571: Why were these fractions not analyzed, sampling issues?

*REPLY: The focus of the overall research project was on the medium to large sized phytoplankton taxa, specifically filamentous, diazotrophic cyanobacteria. To obtain sufficient amounts of phytoplankton cells in this oligotrophic lake, we chose to concentrate up to 10 litres of lake water through a 10 µm plankton net. These microscopic samples were used to verify molecular cyanobacterial markers (phycocyanin and phycoerythrin), whereas chlorophyll-*a *served as a total phytoplankton community proxy. For further details, please see Ehrenfels et al. (2021), Front Environ Sci., 9:277 (*[*https://www.frontiersin.org/article/10.3389/fenvs.2021.716765*](https://www.frontiersin.org/article/10.3389/fenvs.2021.716765)*).*

l. 580-581, Can’t there be competiotion from the nano- and picc phytoplankton also?

*REPLY: Yes, indeed. We discuss this in L.569-578.*

l. 607-620 Feels reduntant in this context.

*REPLY: There are different mechanisms underlying the C and N isotopic composition of POM, respectively. Thus, we think that discussing the δ^15^N-POM in the context of the hydrodynamic regime and associated N sources of the primary producers deserves a paragraph here.*

l. 702, “phenotypic changes” is a bit odd in this context. From this study there is no indication (not studied) fish make any phenotypic changes (as number of gill rakers or gut length) or behavior (vertical migration, diet changes), all changes may just be an effect of the change in isotope signal of prey.

*REPLY: We rephrased this sentence and now explicitly refer to the phenotypic parameters studied in this paper. The respective part reads now as follows: “Despite the absence of pronounced spatial genetic structure in either of the sardine* (Junker et al., 2020; De Keyzer et al., 2019) *or* Lates *species* (Rick et al., 2021)*, phenotypic traits, such as diets and lipid contents, may vary between regional fish populations in response to regionally different environments…”.*

**Reviewer #3:** The authors provide seasonal isotopic data of two sites of Lake Tanganyika to study the regionality of the fish population. Authors have carried out massive sampling and provide detailed analysis based on the carbon and nitrogen isotopes. My main suggestion to the authors is to provide a picture of the whole food web in Lake Tanganyika based on the literature (add this as figure 1).

*REPLY: Done.*

Moreover, authors compare dry and rainy season and it would be interesting to know how phytoplankton communities are assumed to differ based on the literature. The introduction would greatly benefit if the introduction could describe whole food web starting from the phytoplankton – zooplankton -fish including a description of the main species. One could assume that upwelling is an important occasion for diatoms and diatom-based food web.

*REPLY: Done. We agree that a more precise depiction of the food web (both in text and graphically) helps to better understand the paper. We have thus added a conceptual figure to the introduction (see figure 1), briefly describe the major trophic relationships (L.57-62), and have included a sentence regarding the phytoplankton community composition (L.107-109).*

In the result section authors could also provide picture(s) of carbon and nitrogen values of all studied food web components of both sites and seasons. This could help readers understand seasonality and site impact at the whole food web level and to understand if the regionality is only related to the specific fish species or can we see systematical differences at the different trophic levels between north and south and seasonal effect. Or could you put whole of your data (isotopes, biotic and abiotic measurements) to the multistatistical analysis (PCA, NMDS) and show what is actually happening on these two seasons and sites.

*REPLY: Done. We compiled the data in summary tables and performed a statistical analysis across the data set (see figures 7, S7, and S8 as well as tables S1 and S2). We believe that this analysis not only improves understandability, but also strengthens the conclusions we drew from the data.*

*Only five sites with a high overlap across all variables of the data for both of the sampling campaigns. With so few “replicates”, we chose not to do a PCA or NMDS, and instead calculated rank-based correlation matrixes (Fig 6 and S10 Fig). We added our statistical approach to the methods section “2.9 Data analysis”:*

*“To test to what extent the physicochemical and biological variables correlate, we chose the five sites with the highest possible overlap across all variables (Sep/Oct: stations 1, 2, 6, 7, and 9; Apr/May: stations 1, 2, 4, 7, 8). The data represent either depth-integrated values or averages per site. In the resulting data set were 24 gaps compared to a total of 280 data points (10 sites and 28 variables). Gaps at the northern (station 1) or southern (station 9) extremities of the lake were filled by assuming the same value as from the neighbouring site. Other gaps were filled by calculating the average value between the two neighbouring sites (S2 Table). We produced the correlation matrixes using the R package corrplot (70) and calculated the Spearman's rank correlation coefficient (some variables were not normally distributed; Shapiro-Wilk-Test, p < 0.05).”*

*The results are described in section 3.5 and picked up throughout the discussion again.*

Regarding on the nitrogen isotopes I wonder if the authors are aware of how upwelling influence on the nitrogen cycle and the uptake of nitrogen by primary producers (ammonium or nitrate, Bartrons 2009: DOI: 10.5194/bgd-6-11479-2009). This could explain differences in nitrogen values.

*REPLY: Correct, upwelling may alter the availability of different nitrogen sources, such as ammonium or nitrate, to primary producers and thus, affect their resulting δ^15^N value.*

*In the manuscript, we only discuss nitrate and nitrogen fixation as possible new sources of nitrogen, because upwelling was too weak to supply deep-water ammonium into the productive surface waters during our study period. Ammonium concentrations were below limit of detection in the upper 100 m (or deeper) at all stations during both sampling campaigns. We added the figure to the supplements (S3 Fig) and state in the results section that “Ammonium concentrations were below limit of detection in the upper 100 m during both sampling campaigns” (L.361).*

I recommend authors to add water temperature to picture 1 at least assumed range.

*REPLY: Done.*

In the methods you describe that you have used the Folch method for lipid extraction, however, the Folch method uses chloroform, methanol, and water in the proportions 8:4: 3, please check your reference. Secondly, you do not have supernatant in the lipid analysis, but lower phase which includes lipids, and this phase is usually transferred to the new tube.

*REPLY: Done. We adapted the reference to “Chen, I. S., Shen, C. S. J., & Sheppard, A. J. (1981). Comparison of methylene chloride and chloroform for the extraction of fats from food products. Journal of the American Oil Chemists’ Society, 58(5), 599-601”. We furthermore clarified that the lower phase (and not the supernatant) were transferred to the new tube.*

In figure 3 you could provide letter on which site station is located e.g. station 1 (N) or station 8 (S).

*REPLY: Done. We followed through also for supplementary figure S5 (formerly figure 4).*

In line 541 you say that lipid content reduced by 43 to 45%, however, I would keep it more informative if you could provide real values, e.g. lipid content reduced from x to y.

*REPLY: Done.*

**References**

Coulter, G. W.: Population changes within a group of fish species in Lake Tanganyika following their exploitation, J. Fish Biol., 2, 329–353, 1970.

Coulter, G. W.: Lake Tanganyika and its life., British Museum of Natural History, London and Oxford University Press, Oxford., 1991.

Junker, J., Rick, J. A., Mcintyre, P. B., Kimirei, I., Sweke, E. A., Mosille, J. B., Wehrli, B., Dinkel, C., Mwaiko, S., Seehausen, O. and Wagner, C. E.: Structural genomic variation leads to genetic differentiation in Lake Tanganyika’s sardines, Mol. Ecol., 29, 3277–3298, doi:10.1111/mec.15559, 2020.

De Keyzer, E. L. R., De Corte, Z., Van Steenberge, M., Raeymaekers, J. A. M., Calboli, F. C. F., Kmentová, N., N’Sibula Mulimbwa, T., Virgilio, M., Vangestel, C., Mulungula, P. M., Volckaert, F. A. M. and Vanhove, M. P. M.: First genomic study on Lake Tanganyika sprat Stolothrissa tanganicae: A lack of population structure calls for integrated management of this important fisheries target species, BMC Evol. Biol., 19(1), 1–15, doi:10.1186/s12862-018-1325-8, 2019.

Matthes, H.: Preliminary investigations into the biology of the Lake Tanganyika Clupeidae, Fish. Res. Bull. Zambia, 4, 39–45, 1967.

Rick, J. A., Junker, J., Kimirei, I. A., Sweke, E. A., Mosille, J. B., Dinkel, C., Mwaiko, S., Seehausen, O. and Wagner, C. E.: The Genetic Population Structure of Lake Tanganyika’s Lates Species Flock, an Endemic Radiation of Pelagic Top Predators, J. Hered., esab072, doi:10.1093/jhered/esab072, 2021.
