# Supplementary material for: Isotopic signatures induced by upwelling reveal regional fish stocks in Lake Tanganyika
Source: PLoS One. 2023 Nov 8;18(11):e0281828. doi: 10.1371/journal.pone.0281828 (PMC10631627; doi:10.1371/journal.pone.0281828)
Supplement: Attachment — Submitted filename: response_to_reviewers_ehrenfels.docx [file pone.0281828.s017.docx]

Below is a point-by-point response to all reviewer comments. Changes in the manuscript are documented in the mark-up mode version of the revised manuscript.

**Reviewer #2**: Although there have been several improvements of the manuscript I still find some weaknesses. In general, I still find especially the result section rather long and inconclusively written with a lot of detailed numbers that I think could be referred to figures (or Tables) instead. There is also a mix of presenting significant and non-significant results (compare l. 376, 391).

**Reply**: Done. We have streamlined the respective parts and present them in a more uniform fashion. Due to the small sample size of the planktonic parameters, we also mention insignificant differences in the text, especially when they follow systematic patterns.

I think for example you could remove or substantially shorten

- 342-353: **Reply**: we shortened this part;
- 355-363: **Reply**: we shortened this part;
- 384-386: **Reply**: we removed this part;
- 391-403: **Reply**: we shortened this part;
- 433-448: **Reply**: we shortened this part;
- 462-474: **Reply**: we shortened this part;
- 482-486: **Reply**: we removed this part.

Maybe I was a bit unclear in my previous review or I misunderstand the interpretations of the result, but I cannot see that one of the main conclusions; “regional fish populations” hold based on this data. To expand my thoughts on this a bit. In the introduction it is referred to a study by Logan et al that “…isotopic study of fish and their surrounding food web along a geographical gradient can reveal regional population isolation if environmental differences among sites translate into divergent isotopic signatures of regional or local fish populations”. This is true but the cited study consider a global study comparing three different tuna-species over 16 years, which your study is not even close to. Importantly, they study GRADIENTS in isotopic signals whereas your paper focus on north and south samples, not a gradient, compare with Fig. 8 & 9 in Logan et al. In your study, if you assume all fish move completely random (i.e. no regional/spatial structure) fish will pick up signal of the food-web where it feeds, i.e. the differences in Fig. 4 are reflected in the fish in Fig. 5, or? Maybe I miss something important as I find it hard to pick out the important information in the result section, but I just don’t get how random movements will differ from movements with some “center of gravity”, unless you think fish traverse ~450 km (4°?) within a couple of weeks. Some clearly stated hypothesis of what is expected if there are “regional fish population” or not is necessary, especially as you don’t have extensive gradients as in Logan et al.

**Reply:** Done. We agree that our chosen terminology was not ideal, because it was not precisely congruent with the standard biological definition of what a population is. Following the detailed explanations of the reviewer, we have made three substantial changes.

First, we rephrased “regional populations” throughout the entire manuscript. We now refer to “regional fish stocks”, which to the best of our knowledge is a term with a less preoccupied meaning. Alternatively, we also changed the wording simply to “samples”, “specimens” or entirely rephrased the respective text.

Second, to describe the restricted movement with the studied period (two seasons), we have introduced the term “regionally sessile”. We explain what we mean by this term and formulate a testable hypothesis in the final paragraph of the introduction (L. 167-172): *“Assuming fish were regionally sessile within the investigated time period, instead of moving randomly across the lake, we expect fish to reflect the isotope signatures from the planktonic base of the food web. Thereby, when we refer to “regionally sessile”, we do not distinguish between random movement with a geographic reach equal or smaller than the basin-scale and movement with a geographic centroid related to population dynamics.”*

Third, we changed the title accordingly, which now reads as follows: “*Pelagic fish in Lake Tanganyika are regionally sessile but lack local adaptation”*.

Moreover, in the Conclusions it says “…regional fishery management strategies should consider including basin-scale quotas.” But what is basin-scale, from the profile in Fig. 2 there are no apparent “basins”? Based on your results, how many basins and where should the borders be according to you?

**Reply:** Done. In the literature, Lake Tanganyika is characterized by three basins, of which we compare the two extremes with each other, i.e. the north and south basins. We added borders to Fig. 2 a to visualize which stations relate to which basin. In the text, we have now defined that we use the term *“region/regional”* as a synonym for *“basin/basin-scale”*. We clarified this upon first mention of the term in the introduction (L. 104) and again in the head of the discussion (L. 533): *“…regional (i.e. basin-scale)…”.* Moreover, to achieve more consistency, we changed *“spatial”* to *“regional”* throughout the manuscript, whenever it was used in this context.

I find the study to be interesting also without any conclusions about fish population structure. However, I think the current focus on regional fish populations may be misleading.

**Reviewer #3**: Your revised version of the manuscript has improved especially illustration of figures 1-3 is now much better. You have added some correlation analysis, but you have skipped the main point for multivariate statistical analysis, which is to shorten and clarify the message of your manuscript. So, the point is to remove single isotope pictures (Fig. 4 c, d, g, h, I, j and Fig. 5) and have one stable isotope picture in PCA or NMDS plot which can clearly demonstrate how different site and season influence on the isotope value and food web structure. So, I still urge you to put all stable isotope data in PCA or NMDS and show your stable isotope data as one figure. See e.g. figure 4 in Ramos et al. 2009, who also published their paper in PlosOne doi:10.1371/journal.pone.0006236.

**Reply:** Done. While we value this constructive review, here we feel that the reviewer imposes a very detailed prescription how we must present our data. Nonetheless, in order to improve the presentation of the data, we added a PCA (Fig. 8) to demonstrate the influence of latitude and season on the isotopic signature of the food web, as suggested.

We did not understand the benefit of doing a PCA with only two dimensions (δ^13^C and δ^15^N). The exemplary figure from Ramos et al. includes three isotope ratios. We have thus included the C:N ratio in our PCA analysis. This analysis supports the main result: δ^13^C explains most of the variation between samples from the north and south during Sep/Oct.

We kept figures 4 and 5 to illustrate the key contrasts, i.e. north-south differences of the two isotope ratios between the two seasons and different food web members, also in the conventional isotope biplot fashion. We believe it is essential for the reader’s comprehension to view the original data points in the two dimensional isospace. To better link the data from planktonic samples with the tissue samples, we have added panels for POM and zooplankton to figure 5.

Finally, we cite Ramos et al. (2009) as an example for how biogeochemical markers from different water masses can be used to delineate migratory and residency patterns in pelagic animals.
