# Supplementary material for: Isotopic signatures induced by upwelling reveal regional fish stocks in Lake Tanganyika
Source: PLoS One. 2023 Nov 8;18(11):e0281828. doi: 10.1371/journal.pone.0281828 (PMC10631627; doi:10.1371/journal.pone.0281828)
Supplement: Attachment — Submitted filename: response_to_reviewers_ehrenfels.docx [file pone.0281828.s018.docx]

Below is a point-by-point response to all reviewer comments. Changes in the manuscript are documented in the mark-up mode version of the revised manuscript.

**Reviewer #2**: Overall I think the authors have improved the writing, terminology and structure of the manuscript. I have some smaller issues but I think they can easlily be addressed by the authors.

1. I don't think the change of title was very successfull, don't really know what motivated this but think the former title is more informative.

*Reply:* *Done. We have changed the title back to the original version, but replaced “populations” by “stocks” (“Isotopic signatures induced by upwelling reveal regional fish stocks in Lake Tanganyika”). The term “populations” was one of the previous key criticisms.*

2. I'm skeptic to 'regional sessile' as it imply they are some how fixed to some substrate. Maybe something along regional forage grounds that you use on l. 39 or so is better. As I understand actual spawning araes are not identidied?

*Reply: We agree with the reviewer’s concern here about the term “sessile”. Thus, we have reverted our wording in describing these stocks as “regional”.*

*We do not have information about spawning areas. This would be fantastic to have, but unfortunately these detailed aspects of the life history of these fishes remain unknown. However, as we exclude juvenile fish from our analyses (see section 2.9), we do not believe that our results are biased by spawning ground location. In support of this argument, north-south differences in δ^13^C during the dry season were consistent across all trophic levels. Furthermore, differences with respect to spawning ground preferences exist among the studied fish species. In brief,* Stolothrissa *and* Lates stappersii *spawn offshore, whereas* Limnothrissa *and the three larger* Lates *species,* L. microlepis, L. mariae, *and* L. angustifrons *nearshore (L. 64).*

3. To that respect, could region and basins be defined? it seems like the southern basin is >7 degree south, or? but what is northern basin/stock? On the map there is a central basin, but there were no fish samples from this basin if I got it right. So should this be a basin/region on its own or part of souty/north stock?

*Reply:* *Done. We have clarified the synonymous use of region and basin upon first mention of the term in the introduction (L. 108) and again in the head of the discussion (L. 539): “…regional (i.e. basin-scale)…”. The basins are clearly defined in figure 2.*

*In the methods section and captions of the respective figures, we additionally communicate from which stations the analyzed samples were taken. See e.g. L 314: “For comparing the isotopic composition and the C:N ratios of fish samples between different regions, we selected individuals from sites where samples were available from both campaigns (north: landing sites adjacent to stations 1 and 2; south: landing sites adjacent to stations 7 and 9).”*

*All samples, including those from the central basin displayed in the supplements (S4 Figure). Samples from the central basin were also included in the correlation analyses to show the gradual shifts from north to south in the dry season.*

*Since we do not assume that the fish stocks are populations with a geographic center, we assume that the central basin is a transition zone between north and south with respect to isotopic signal of the pelagic fish. In line with this, the figure below (now added as S5 Fig) illustrates that our samples from the central basin are nested within the distributions from the north and south. With respect to the hydrodynamics, we think that the central basin is more similar to the north basin, whereas the strong upwelling in the south makes the southern basin exceptional.*

*We did not include samples from the central basin in the illustration of our key findings (Figure 5), because we did not have samples from the same size range or station from both campaigns.*

*Overall, focusing on north and south helped to simplify and clarify the message.*

4. On l. 40-41 you write "...fish reside in a region for a season or longer." You should clarify that you have studied seasonal variation, what happens in a longer run you don't know. To me it seems fully possible that they aggregate in the south during the productive upwelling but then disperse over the lake for the rest of the year. There are many examples of fish where different stocks (spawning units) mix in a productive area for foraging and then return to more "native" areas for spawning (salmon maybe being the most extreme example). So you don't know what the long term distribution look like.

*Reply:* *Done. We agree that this is an important message to be clear on. We clarify that we studied seasonal data in the abstract (L. 34), the introduction (L. 159, 162, 167), the methods (L. 181-190), and the discussion (e.g. L. 547-556) sections. We agree that we cannot know what happens on a multiannual time scale. Thus, we deem it necessary to communicate this uncertainty explicitly. Therefore, we added terms like “at least on a seasonal scale” or “for a season or longer”. If we wrote that “the fish reside in a region for a season.”, we would implicitly exclude the potential for longer residence times. We communicate this more clearly now in the respective discussion section (L. 703): “i.e. the fish move randomly across the lake on long term generational time scales. At least on a seasonal time scale, however, the fish reside in a region, even though the exact time scale of restricted movement is unclear.”*

5. l. 127, exchange effects for barriers

*Reply:* *Done.*

6. l. 754-756: It may be the other way around, as the environment may be rather homogenous with, similar prey (and predators?) there are only very weak barriers to gene flow.

*Reply:* *We agree that the interaction can go both ways. Thus, we formulated this final statement as a hypothesis.*

*Our reasoning for stating the hypothesis as we have is that for pelagic species there would be no physical barrier to the gene flow as long as the basins are hydrologically connected, even if the environmental differences between north and south were far greater.*

*Second, previous studies in Lake Tanganyika revealed that differences in turbidity as well as the relative species composition of the plankton and nekton exist between the basins (e.g. Descy et al., 2005; Hecky and Kling, 1981; Plisnier et al., 2009). In line, Mannini et al. (1999) found that predatory fish were specialized on different prey items in the different basins.*

*In our study, we could not resolve subtle dietary differences with our methodology (bulk δ^15^N). Based on our data, we suggest that the trophic level does not vary substantially, yet the actual prey items may be very different. These is indeed an important distinction. Thus, we have changed “diets” to “trophic level”, wherever applicable.*

**References**

Coulter, G. W.: Population changes within a group of fish species in Lake Tanganyika following their exploitation, J. Fish Biol., 2, 329–353, 1970.

Coulter, G. W.: Lake Tanganyika and its life., British Museum of Natural History, London and Oxford University Press, Oxford., 1991.

Descy, J. P., Hardy, M. A., Sténuite, S., Pirlot, S., Leporcq, B., Kimirei, I., Sekadende, B., Mwaitega, S. R. and Sinyenza, D.: Phytoplankton pigments and community composition in Lake Tanganyika, Freshw. Biol., 50(4), 668–684, doi:10.1111/j.1365-2427.2005.01358.x, 2005.

Hecky, R. E. and Kling, H. J.: The phytoplankton and protozooplankton Lake Tanganyika: Species composition, biomass, chlorophyll content, and spatio-temporal distribution, Limnol. Ocean., 26(3), 548–564, doi:10.4319/lo.1981.26.3.0548, 1981.

Mannini, P., Katonda, K. I., Kissaka, B. and Verburg, P.: Feeding ecology of Lates stappersii in Lake Tanganyika, in From Limnology to Fisheries: Lake Tanganyika and Other Large Lakes, edited by O. V. Lindqvist, H. Mölsä, K. Salonen, and J. Sarvala, pp. 131–139, Springer Netherlands., 1999.

Matthes, H.: Preliminary investigations into the biology of the Lake Tanganyika Clupeidae, Fish. Res. Bull. Zambia, 4, 39–45, 1967.

Plisnier, P. D., Mgana, H., Kimirei, I., Chande, A., Makasa, L., Chimanga, J., Zulu, F., Cocquyt, C., Horion, S., Bergamino, N., Naithani, J., Deleersnijder, E., André, L., Descy, J. P. and Cornet, Y.: Limnological variability and pelagic fish abundance (Stolothrissa tanganicae and Lates stappersii) in Lake Tanganyika, Hydrobiologia, 625(1), 117–134, doi:10.1007/s10750-009-9701-4, 2009.
